# Influence of the COVID-19 Pandemic on Clinical Trial Discontinuation in Anesthesiology: Cross-sectional Analysis

**DOI:** 10.2196/34936

**Published:** 2022-04-05

**Authors:** Brett D Traxler, Brayden M Rucker, Mary C Greenough, Nicholas B Sajjadi, Micah Hartwell

**Affiliations:** 1 Office of Medical Student Research College of Osteopathic Medicine Oklahoma State University Center for Health Sciences Tulsa, OK United States; 2 Office for Research Development and Scholarly Activity School of Community Medicine University of Oklahoma Tulsa, OK United States; 3 Department of Psychiatry and Behavioral Sciences College of Osteopathic Medicine Oklahoma State University Center for Health Sciences Tulsa, OK United States

**Keywords:** clinical trials, anesthesia, anesthesiology, COVID-19, pandemic, perioperative care, lockdown

## Abstract

**Background:**

The COVID-19 pandemic drastically altered perioperative medical practice owing to safety concerns, postponing elective or nonemergent procedures, supply chain shortages, and reallocating perioperative staff to care for patients with COVID-19. However, the impact of the pandemic on the conduct on anesthesiology clinical research is unknown.

**Objective:**

The primary objective was to quantify the magnitude of the COVID-19 pandemic’s impact on anesthesiology clinical research.

**Methods:**

We performed a systematic search using ClinicalTrials.gov to identify clinical trials related to the practice of anesthesiology. We screened trials with status updates from January 1, 2020, through October 1, 2021, to capture trials potentially affected by the COVID-19 pandemic by the time of our search. Investigators screened for relevant studies and extracted trial characteristics along with the reason for discontinuation reported on the clinical trial registry.

**Results:**

A total of 823 clinical trials met inclusion criteria, and 146 clinical trials were discontinued within the designated date range. In total, 24 (16.4%) of the 146 clinical trials were halted explicitly owing to the COVID-19 pandemic. A significant association existed between trial enrollment numbers and the likelihood of discontinuation due to the COVID-19 pandemic, as larger trials were more likely to be disrupted (*z*=–2.914, *P*=.004).

**Conclusions:**

The COVID-19 pandemic is reportedly associated with the discontinuation of anesthesiology-related clinical trials. With the uncertain course of the COVID-19 pandemic, developing anesthesia trial protocols to help minimize social interaction and prevent premature trial disruption are imperative.

## Introduction

Clinical trials are at the forefront of modern medicine and evidence-based care, as they provide novel diagnostic tools and interventions for a variety of conditions [1-3]. Unfortunately, the COVID-19 pandemic has significantly disrupted clinical trial conduct and hindered trial accessibility and overall development owing to public safety measures including lockdowns and mandatory closures [4-6]. Moreover, updated guidelines from the US Food and Drug Administration (FDA) encouraged trialists to carefully consider whether to continue studies in light of risks associated with the COVID-19 pandemic and to either accommodate trial design to mitigate risk or to discontinue studies indefinitely [7,8]. Clinical trialists also faced recruitment difficulties during the pandemic, as referring physicians were reallocated to assist with pandemic efforts [9]. The surgical specialties have also been impacted, as many procedures deemed elective or nonemergent were canceled, thereby reducing time in the operating room [9]. Changes to clinical practice during the pandemic had direct consequences on the conduct of clinical trials [5].

Anesthesiologists have played a vital role in the COVID-19 pandemic response, specifically regarding airway management and ventilatory support [10]. Many patients with COVID-19 develop profound hypoxemia, pulmonary infiltrates, and altered lung function requiring intubation and ventilatory support [11]. A multicenter study conducted over 3 months reported that over 80% of patients with confirmed COVID-19 were intubated [11]. Owing to increased aerosolization of respiratory secretions during bag-mask ventilation, many institutions have implemented rapid sequence induction for all patients requiring ventilatory support to prevent the potential spread of COVID-19 [12,13]. As the pandemic continued, the number of patients requiring intubation—along with prolonged intubation times—increased significantly, leading to ventilator shortage [14]. Moreover, the increased demand for anesthetic equipment required for intubation has led to a downstream shortage of supplies for elective cases, placing patients who do not have COVID-19 at a disadvantage to receiving adequate care [15]. The clinical practice of anesthesiologists changed drastically during the pandemic [16], and together with the aforementioned changes in clinical trial conduct, it is likely that ongoing anesthesiology clinical trials were disrupted during the pandemic. Given the important role of perioperative medicine, anesthesiologists will continue to rely on findings from clinical trials to stay up to date with novel interventions and therapies, and interruptions to clinical research may have important implications. Therefore, the primary objective of this study is to highlight the impact of the COVID-19 pandemic on the progress of clinical trials related to anesthesiology.

## Methods

### Search Strategy

We conducted a systematic search of ClinicalTrials.gov, an international registry of both privately and publicly funded clinical studies, for trials related to anesthesiology on October 2, 2021 [17]. Our search string included the following terms: *Anesthesia*, *Anesthesiology*, *Anesthesiologist*, *General Anesthesia*, *Standard Induction of General Anesthesia*, *Mask Ventilation*, *Laryngeal Mask Airway*, *Monitored Anesthesia Care*, *Endotracheal Intubation*, *Awake Fiberoptic Intubation*, *Left-sided Double Lumen Tube*, *Wire Cricothyroidotomy*, *Spinal Anesthesia*, *Lumbar Epidural*, *Regional Anesthesia*, and *Peripheral Nerve Block*. To retrieve all trials potentially impacted by the COVID-19 pandemic, we used the date range of January 1, 2020, through October 1, 2021. The search string is presented in Textbox 1.

**Search string for clinical trials.**

ClinicalTrials.gov:

Anesthesia OR Anesthesiology OR Anesthesiologist OR General Anesthesia OR Standard Induction of General Anesthesia OR Mask Ventilation OR Laryngeal Mask Airway OR Monitored Anesthesia Care OR Endotracheal Intubation OR Awake Fiberoptic Intubation OR Left-Sided Double Lumen Tube OR Wire Cricothyroidotomy OR Spinal Anesthesia OR Lumbar Epidural OR Regional Anesthesia OR Peripheral Nerve Block | Recruiting, Active, not recruiting, Enrolling by invitation, Suspended, Terminated, Withdrawn Studies | Interventional Studies | Phase Early Phase 1, 1, 2, 3, 4 | Last update posted from 01/01/2020 to 10/01/2021

### Eligibility Criteria

Trials were included on the basis of the following criteria: (1) the study is relevant to the clinical practice of anesthesiologists for use in perioperative care including induction, sedation, emergence, analgesia, hemodynamic stability, oxygenation, pain management, and complications secondary to anesthetic methods; (2) the study is interventional in nature; (3) the study status is ongoing (recruiting, active but not recruiting, enrolling by invitation) or discontinued (suspended, withdrawn, or terminated); and (4) the study is in any phase (I, II, III, and IV). Trials that did not meet all of the inclusion criteria were excluded from the analysis.

### Data Extraction

Resulting trials from the search strategy were extracted for trial status, condition treated, enrollment number, funding, study type, study design, last update posted date, and trial location. We screened trials for relevance to the field of anesthesiology in accordance with the “conditions treated.” Any studies irrelevant to the field were excluded. Two authors (BT and BR) extracted reasons for discontinuation provided on the ClinicalTrials.gov website in a blinded, duplicate manner. Trials that explicitly stated “COVID-19” in the “Recruitment Status” box on ClinicalTrials.gov as a reason for discontinuation were coded as such. We additionally extracted trial intervention of these studies. Trials that failed to mention the COVID-19 pandemic as a reason for discontinuation were coded for the reason provided. Trials that did not specify a reason for discontinuation were coded as “not provided.”

### Statistical Analysis

To determine any significant differences in enrollment between trials discontinued owing to the COVID-19 pandemic versus all other discontinued trials, we used a Mann-Whitney *U* test, which was the preferred test because enrollment numbers were nonnormally distributed among our sample trials. Fisher exact tests were used to determine associations between trials halted owing to the COVID-19 pandemic with the funding source and trial location (US-based and non–US-based trials). Studies that listed multiple sites were coded as US-based if any of the sites were US locations. The funding source was coded as either *US Government* (US Federal agency, Veterans Affairs, Department of Defense, etc), *Industry* (if any industry involvement was reported), or *Other* for registered clinical trials receiving funding from neither industry nor government. *Other* is a “funded by” category that investigators can select on ClinicalTrials.gov, and we applied the term as defined on ClinicalTrials.gov. If a study included *US Government* and *Industry*, it was coded as *US Government* owing to superseding reporting guidelines, with the same hierarchy for studies with *Industry* and *Other*.

Statistical analyses were performed using Stata (version 16.1; StataCorp).

### Ethical Considerations

Our study did not meet the criteria for human subject research according to the institutional review board [18]. ClinicalTrials.gov is an open source database that does not contain identifiable private information. Therefore, using the 2018 flow chart provided by the US Department of Health and Human Services [19], it was determined that our research did not involve human subjects and that the Protection of Human Subjects under United States Law (1974), the Code of Federal Regulations Title 45: Public Welfare, part 46 (45 CFR 46) did not apply.

## Results

### Study Characteristics

Our original search string returned 1595 trials, and 823 were included after screening for relevance to our search. Among the 823 included trials, the median enrollment was 80 (IQR 40-133) with a total of 160,021 participants (range 0-2000). By funding source, 52 (6.3%) were categorized as *Industry*, 20 (2.4%) as *US Government*, and 751 (91.3%) as *Other.* By trial status, 70 (8.5%) were categorized as active, 39 (4.7%) were enrolled by invitation, 568 (69.0%) were recruiting, 19 (2.3%) were suspended, 76 (9.2%) were terminated, and 51 (6.2%) were withdrawn. By the phase of trial conduction, 82 (10.0%) were in phase 1, a total of 108 (13.1%) and 185 (22.5%) were in phases 2 and 3, respectively, and 448 (54.4%) were in phase 4.

### Discontinued Trials

Of the 823 included trials, 146 (17.7%) were discontinued between January 1, 2020, and October 1, 2021. Of the discontinued trials, the median enrollment was 7 (IQR 0-50) with a range of 0 to 407 participants. A total of 5816 participants were involved in prematurely discontinued studies. By funding source, 16 (11.0%) were categorized as *Industry*, 3 (2.1%) as *US Government*, and 127 (87.0%) as *Other*. A total of 54 (37.0%) trials were conducted in other countries and 92 (63.0%) were conducted in the United States. By study design, 16 (11.0%) were nonrandomized, while 130 (89.0%) were randomized, and 103 (70.6%) included masked study participants, while 43 (29.5%) included unmasked participants. Regarding study intervention, 3 (2.1%) were categorized as *Device*, 122 (83.6%) as *Drug*, 20 (13.7%) as *Procedure*, and 1 (0.7%) as *Other*. By trial status, 19 (13.0%) were categorized as suspended, 76 (52.1%) as terminated, and 51 (34.9%) as withdrawn. The reported reasons for discontinuation were as follows: recruitment and enrollment (n=39, 26.7%), sponsor-related (n=2, 1.4%), safety and efficacy (n=8, 5.5%), PI-related (n=15, 10.3%), funding and resources (n=10, 6.8%), design-related (n=23, 15.8%), lack of approval (n=7, 4.8%), not provided (n=10, 6.8%), other (n=8, 5.5%), and owing to the COVID-19 pandemic (n=24, 16.4%).

### COVID-19–Related Discontinuation

Of the 146 discontinued trials, 24 (16.4%) were halted explicitly owing to the COVID-19 pandemic as stated on ClinicalTrials.gov. These trials had a median trial enrollment of 32.5 (IQR 19.5-85.5) with a range of 0-400. By trial status, 10 (41.7%) were suspended, 9 (37.5%) were terminated, and 5 (20.8%) were withdrawn. The majority of the trials (20, 83.3%) were found to have a primary intervention of *Drug* and 4 (16.7%) had *Procedure.* By study design, 23 (96.0%) were randomized, while 1 (4.0%) was nonrandomized. A total of 18 (75.0%) trials included masked participants, while 6 (25.0%) included unmasked participants. By funding source, 2 (8.3%) trials were funded by *Industry,* 1 (4.2%) was funded by the *US Government,* and 21 (87.5%) were funded by *Other* sources*.* A total of 6 (25.0%) were performed internationally, while 18 (75.0%) were performed in the United States.

### Associations

We found significant associations between termination reasons (COVID-19 vs non–COVID-19) and trial status with 52.6% (10/19) of trials suspended owing to the COVID-19 pandemic, while 11.8% (9/76) of studies were terminated owing to the COVID-19 pandemic, and 9.8% (5/51) trials were withdrawn owing to the pandemic (Table 1). Furthermore, the Mann-Whitney *U* test showed a significant difference in enrollment between trials discontinued owing to the COVID-19 pandemic (median 32.5, IQR 19.5-85.5) and those discontinued owing to non–COVID-19 reasons (median 5, IQR 0-35; *z*=–2.914; *P*=.004).

**Table 1:** Associations among reasons for discontinuation and trial characteristics.

Characteristics		Does not explicitly state COVID-19–related reasons (n=122)	Explicitly states COVID-19–related reasons (n=24)	Total	Chi-square (*df*)		*P* value	
**Status, n (%)**						20.9 (2)		<.001
	Suspended	9 (6.2)	10 (6.9)	19 (13.0)				
	Terminated	67 (46.0)	9 (6.2)	76 (52.1)				
	Withdrawn	46 (31.5)	5 (3.4)	51 (34.9)				
**Intervention, n (%)**						1.0 (3)		.81
	Device	3 (2.1)	0 (0)	3 (2.1)				
	Drug	102 (69.9)	20 (13.7)	122 (83.6)				
	Procedure	16 (11.0)	4 (2.7)	20 (13.7)				
	Other	1 (0.7)	0 (0)	1 (0.7)				
**Randomization, n (%)**						1.3 (1)		.26
	Not randomized	15 (10.3)	1 (0.7)	16 (11.0)				
	Randomized	107 (73.3)	23 (15.8)	130 (89.0)				
**Study design, n (%)**						0.3 (1)		.60
	Masked	85 (58.2)	18 (12.3)	103 (70.6)				
	Unmasked	37 (25.3)	6 (4.1)	43 (29.5)				
**Funding, n (%)**						0.8 (2)		.67
	Industry	14 (9.6)	2 (1.4)	16 (11.0)				
	Other	106 (72.6)	21 (14.4)	127 (87.0)				
	Government	2 (1.4)	1 (0.7)	3 (2.1)				
**Location, n (%)**						1.8 (1)		.18
	Non–US-based	48 (33.0)	6 (4.1)	54 (37.0)				
	US-based	74 (50.7)	18 (12.3)	92 (63.0)				
**Enrollment^a^**						—^b^		.004
	Median (IQR)	5 (0-35)	32.5 (19.5-85.5)	7 (0-50)				
	Range	0-407	0-400	0-407				
	Total	4284	1532	5816				

^a^Mann-Whitney U test, *z*=–2.914.

^b^N/A: not applicable.

## Discussion

### Principal Findings

Our study demonstrated over 1 in 6 anesthesiology-related clinical trials registered on ClinicalTrials.gov at the time of our search were prematurely discontinued as a direct result of the COVID-19 pandemic. Of note, we found a significant association between trial enrollment and the likelihood of reporting discontinuation owing to the COVID-19 pandemic, with larger trials being more likely to be have been discontinued owing to the COVID-19 pandemic. Indeed, over one-fourth of participants involved in discontinued anesthesia clinical trials during the pandemic were from trials that claimed the COVID-19 pandemic as the primary cause for discontinuation.

The considerable rise in social distancing efforts and quarantine guidelines could have limited participant–provider interactions, thus pushing larger trials to halt trial progress until safer conditions could be ensured. From a logistical standpoint, Sathian et al [5] described how the COVID-19 pandemic has disrupted operational planning, activity creation, and decision-making of major clinical trials, causing an overall operational burden to the clinical trial industry. However, properly maintaining clinical trials requires more than just participation. Investigation personnel at clinical sites consistently interact with trial participants, and multiple researchers, fellows, and scientists participate in data collection, some of whom may interact with patients or other members of the care team [20]. Thus, larger trials with more personnel could have been more at risk of premature cessation than smaller trials that were more likely to accommodate social distancing standards. Further, attempting to uphold a trial in a socially distanced world may have outweighed the potential benefit of the trial itself—more trial participants implies more potential exposures to COVID-19. In response to the heightened transmission of COVID-19, the FDA called on researchers and trial sponsors to “determine that the protection of a participant’s safety, welfare, and rights is best served by continuing a study participant in a trial as per the protocol or by discontinuing the administration or use of the investigational product or even participation in the trial” [7]. Discontinuing a study altogether could have been the only way to ensure participant safety among trials with high enrollment. The limitations of person-to-person contact during the pandemic as well as the potential threat of COVID-19 exposure may have predisposed larger anesthesia-related clinical trials to premature discontinuation.

### Future Directions

The need for continued anesthesiology-related research is evident given our study findings. Under current circumstances, unprecedented shortages of ventilators, paralytics, and sedative medications have driven anesthesiologists to practice outside of previous standards of care [21]. For example, Beitler et al [14] proposed using “ventilator sharing” to improve ventilator treatment of patients with COVID-19. Treatment plans and quality care measures are actively evolving in the field of anesthesiology, further pressing the need for upholding anesthesia-related research. A potential solution to maintaining trial progression could be via the establishment of novel communication methods that are more able to withstand a socially distanced society. Wijesooriya et al [22] describes how certain trial activities, such as obtaining informed consent, clinical follow-up, and monitoring parameters such as blood glucose, pulmonary function, and electrocardiography can be performed remotely with new computer models and monitoring tools. Additionally, Bridges et al [23] present the benefits of telehealth for perioperative anesthetic care, as certain undertakings such as mobile phone–based videoconferencing for preoperative and postoperative consultation, at-home data monitoring of fluid status, and anticoagulation, and Bluetooth-connected cardiopulmonary sensors have led to quality improvements within health care systems [24]. However, standardizing data collection methods and maintaining data repositories for anesthesiologists to review remain as challenges to telehealth and should be further explored.

Over 2500 clinical trials were suspended between December 2019 and May 2020, and nearly half of these were suspended owing to the COVID-19 pandemic [25]. Moreover, the *British Journal of Surgery* reported that an estimated 2,367,050 operations per week would be canceled or postponed during the peak 12 weeks of disruption due to the COVID-19 pandemic [26]. Thus, the enormous number of surgeries canceled during the pandemic rendered the conduct of perioperative clinical trials incredibly difficult and may have led to discontinuation of some trials. Our findings reflect the extensive influence of the COVID-19 pandemic on perioperative anesthesia care and highlight an important impact, which may have repercussions on clinical trial progress going forward.

### Strengths and Limitations

First, although over 1 in 6 halted trials from our study explicitly mentioned the COVID-19 pandemic as a reason for discontinuation, it is difficult to determine the exact reasons for discontinuation among the remaining halted trials beyond what was stated directly within the “Recruitment Status” box on ClinicalTrials.gov. Therefore, trials that did not explicitly mention the COVID-19 pandemic as a reason for discontinuation could have potentially been affected by the COVID-19 pandemic, thus underestimating the impact of the pandemic on the conduct of anesthesia-related clinical trials. Second, the nature of our study is cross-sectional and therefore cannot establish causality. Other factors could have contributed to the discontinuation of anesthesiology clinical trials during the pandemic, which were not directly related to the COVID-19 pandemic. Our results should be interpreted with this limitation in mind. Lastly, we did not assess the baseline discontinuation rate of anesthesiology clinical trials prior to the COVID-19 pandemic. Future studies are needed to observe discontinuation rates prior to the pandemic so that proper comparisons can be made. Although we can quantify the trials discontinued during the pandemic, we cannot determine whether the discontinuation differs from previous time intervals. The strengths of our study include the use of ClinicalTrials.gov, which is the largest clinical trial registry. Second, we performed data extraction in a blinded, duplicate manner, which served to reduce systematic error.

### Conclusions

The COVID-19 pandemic has reportedly impacted the progress of anesthesia-related research. Therefore, it is critical to consider further efforts in maintaining trial conduct with the purpose of improving anesthetic care. The value of collective data curation and dissemination to researchers and anesthesia providers has been evident throughout the COVID-19 pandemic. Anesthesia-related research must continue even during difficult times, and the unforeseen end to the COVID-19 pandemic should spark an initiative to incorporate innovative methods for data retrieval and trial conduct within the breadth of anesthesiology.

## Abbreviations

FDA: US Food and Drug Administration
